# What priorities should be considered for Iranian veterans with ankle-foot injuries? A health needs assessment study, 25 years post-conflict

**DOI:** 10.1186/s40779-017-0137-2

**Published:** 2017-09-01

**Authors:** Mostafa Allami, Mohammadreza Soroush

**Affiliations:** Janbazan Medical and Engineering Research Center (JMERC), Tehran, Iran

**Keywords:** Ankle, Foot, Injuries, Veterans

## Abstract

Despite the passage of time, a large number of veterans are still affected by injuries acquired during Iran-Iraq war. In addition to their primary injuries, the majority of veterans also experience difficulty with long-term, secondary effects. Studies have shown that the most common of these include a range of disabilities, pain, and dramatic decline in mental health and quality of life. Improving living conditions and providing rehabilitation services to veterans has always been a main priority of authorities. The goal of this study was to explain the methods and materials with which these priorities were explored.

## Dear editors,

It is estimated that 564,108 people nationwide were injured over the 8 years of the Iran-Iraq conflict (1980˗1988), which corresponds to a rate of 0.7% of the Iranian population [1]. Nearly 100,000 veterans suffer lower extremity injuries, 10,227 of whom have ankle-foot injuries [2]. A large number of war-related traumas are stable, such as amputations, central and peripheral nervous system injuries, and articular degeneration. In the years following the end of the war, a large number of rehabilitation services, including orthotics and prosthetics devices, physical therapy, and drug therapies, have been employed to alleviate pain and unwanted secondary effects and promote quality of life.

The Janbazan Medical and Engineering Research Center (JMERC), with the support of Veterans and Martyr Affair Foundation (VMAF), has conducted several distinct surveys in which groups of veterans with a particular injury were studied. The key aim of these studies was to promote health and help develop veterans’ abilities to manage their personal and social lives. The current study indicated, unexpectedly, that the health problems of veterans with ankle-foot injuries are more serious than those of other veterans.

This health needs assessment study was undertaken by JMERC, with a scientific team that included general practitioners, internists, orthopedists, prosthetic and orthotic specialists, physical therapists and psychologists. Trained experts were responsible for collecting demographic data. The study was carried out in 11 provinces across the country. VMAF invited 2500 veterans with ankle-foot injuries to participate, of whom, 1129 enrolled in this study. Mental health status, quality of life, ability to perform daily living activities, and ability to live independently were evaluated using the Symptom Checklist-90-Revised (SCL-90-R), 36-Item Short Form Survey (SF-36), The Barthel Activities of Daily Living (ADL) Index and the Lawton Instrumental Activities of Daily Living (IADL) Scale survey tools, respectively. Neuromusculoskeletal disorders and neurological lesions were recorded. Any pain in the spine and/or lower limbs was detailed, in addition to defining its primary determinants. Additionally, all types of pain-reducing rehabilitation services received were recorded. The scientific team determined the required rehabilitation services for each case, and appropriate measures were taken at the study site.

## Abbreviations

ADL: Activities of daily living
IADL: Instrumental activities of daily living
JMERC: Janbazan medical and engineering research center
SCL-90-R: The symptom checklist-90-revised
SF-36: 36-Item short form survey
VMAF: Veterans and martyr affair foundation
